# Supplementary material for: Circular EZH2-encoded EZH2-92aa mediates immune evasion in glioblastoma via inhibition of surface NKG2D ligands
Source: Nat Commun. 2022 Aug 15;13:4795. doi: 10.1038/s41467-022-32311-2 (PMC9378736; doi:10.1038/s41467-022-32311-2)
Supplement: Supplementary file 1 — Supplementary Information [file 41467_2022_32311_MOESM1_ESM.pdf]

Supplementary Information

Circular EZH2-Encoded EZH2-92aa Mediates Immune Evasion in Glioblastoma via Inhibition of Surface NKG2D Ligands

Jian Zhong, Xuesong Yang, Junju Chen, Kejun He, Xinya Gao, Xujia Wu,

Maolei Zhang, Huangkai Zhou, Feizhe Xiao, Lele An, Xiuxing Wang, Yu Shi, Nu Zhang

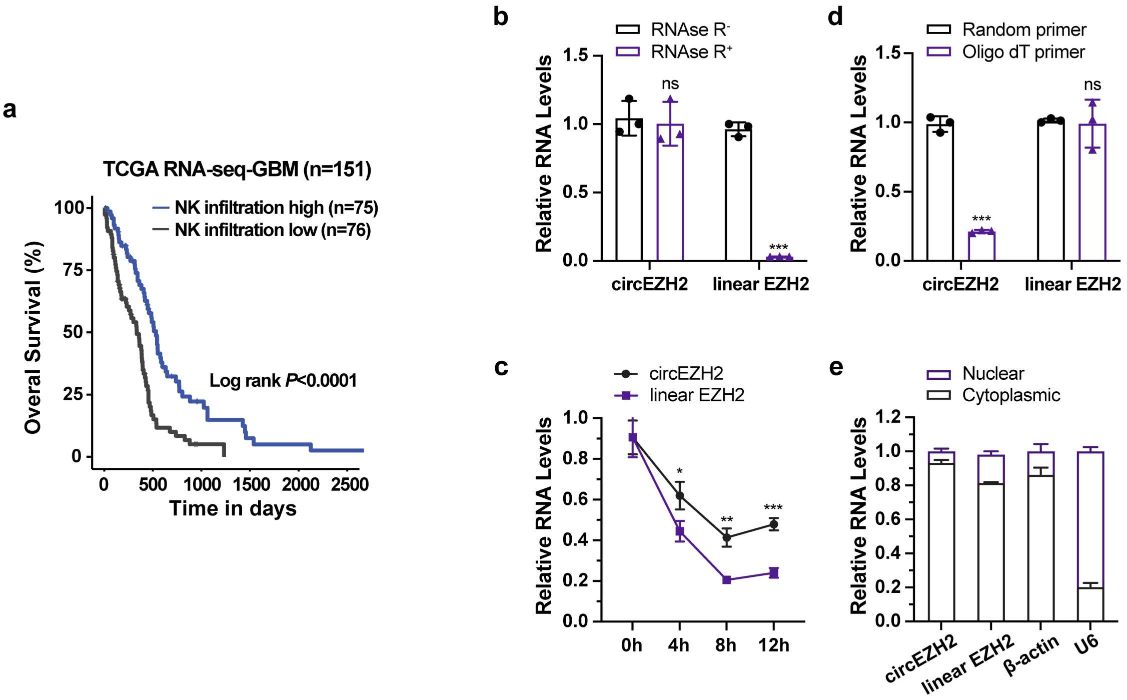

Supplementary Figure 1. NK cell infiltration correlates with the survival of patients with GBM; circular characteristics of circEZH2 (related to Fig. 1)

a. Survival analysis of GBM patients with high and low levels of NK cell infiltration (with the median infiltration score as the cut-off) from a TCGA RNA-seq dataset (n=151). Immune cell infiltration was determined by calculating the gene signature abundance for each cell subtype using an immunophenotyping tool, xCell [http://xCell.ucsf.edu/]. Log-rank test, *P*=8.24e-06.

b. Relative levels of circEZH2 and linear EZH2 after treatment with RNase R. linear EZH2, *P*=5.74e-04.

c. Half-lives of circEZH2 and linear EZH2 mRNA after treatment with actinomycin D (2 μg/ml). 4h, *P*=0.0228; 8h, *P*=0.0014; 12h, *P*=0.0004.

d. Total RNA from MES28 GSCs was reverse transcribed with oligo(dT) primers or random primers. CircEZH2 or linear EZH2 mRNA was detected by qPCR. circEZH2, *P*=1.90e-05.

e. The circEZH2 and linear EZH2 levels were measured in the cytoplasmic and nuclear fractions of MES28 GSCs. β-Actin and U6 RNA served as the cytoplasmic and nuclear controls, respectively.

The data in **(b)-(e)** are presented as the mean ± SD of three independent experiments. Unpaired two-tailed Student's t test was used to determine the significance of differences between the indicated groups. **P*<0.05; ***P*<0.01; ****P*<0.001. Source Data are provided as a Source Data file.

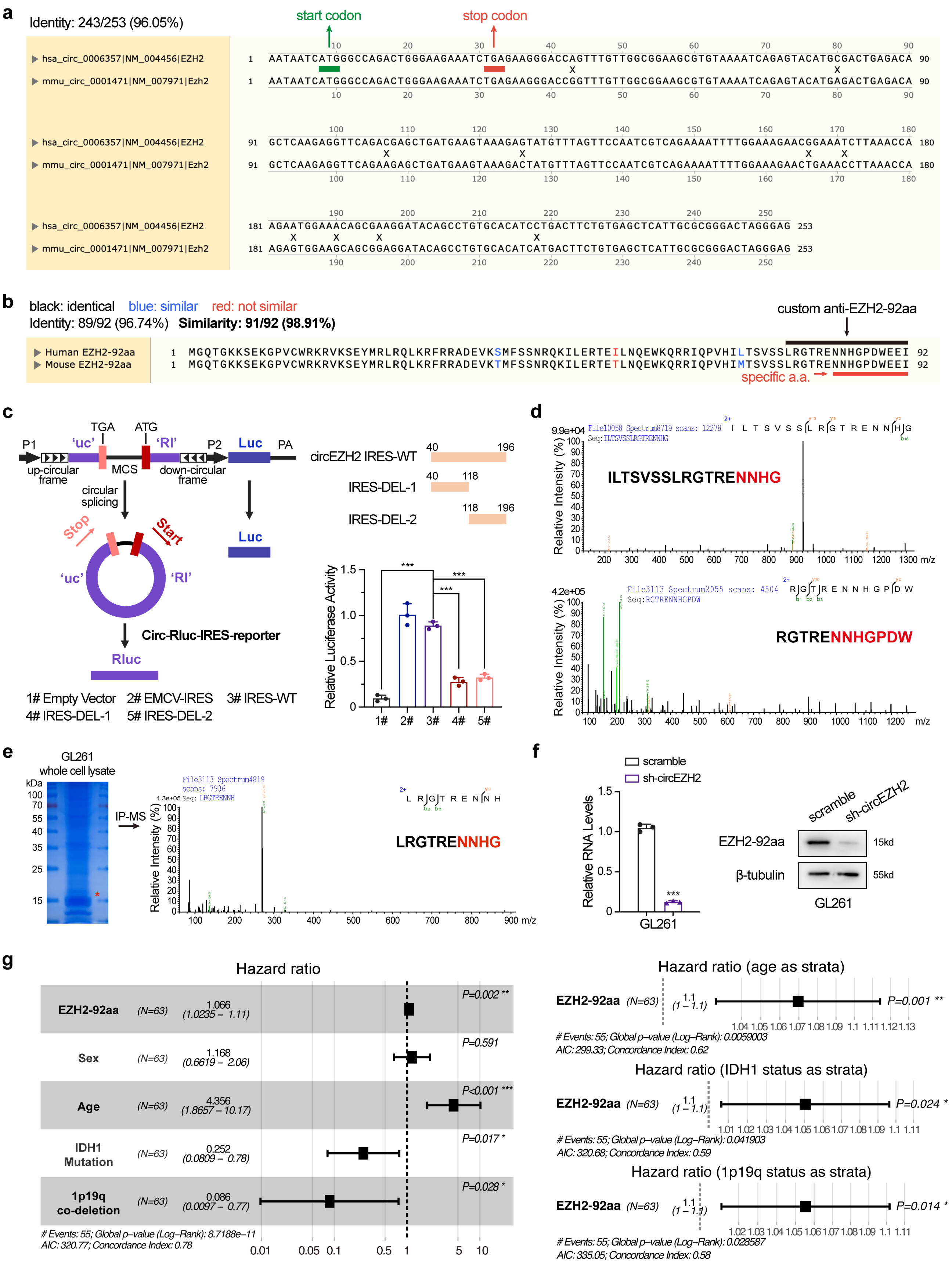
a
Identity: 243/253 (96.05%)
start codon
stop codon
hsa_circ_0006357|NM_004456|EZH2
mmu_circ_0001471|NM_007971|Ezh2
b
black: identical blue: similar red: not similar
Identity: 89/92 (96.74%) Similarity: 91/92 (98.91%)
custom anti-EZH2-92aa
Human EZH2-92aa
Mouse EZH2-92aa
specific a.a.
c
circular splicing
Circ-Rluc-IRES-reporter
circEZH2 IRES-WT
IRES-DEL-1
IRES-DEL-2
1# Empty Vector 2# EMCV-IRES 3# IRES-WT
4# IRES-DEL-1 5# IRES-DEL-2
Relative Luciferase Activity
d
ILTSVSSLRGTRENNHG
RGTRENNHGPDW
Relative Intensity (%)
e
GL261 whole cell lysate
IP-MS
LRGTRENNHG
f
scramble
sh-circEZH2
Relative RNA Levels
EZH2-92aa
β-tubulin
GL261
g
Hazard ratio
Hazard ratio (age as strata)
Hazard ratio (IDH1 status as strata)
Hazard ratio (1p19q status as strata)
EZH2-92aa (N=63) 1.066 (1.0235 – 1.11) P=0.002 **
Sex (N=63) 1.168 (0.6619 – 2.06) P=0.591
Age (N=63) 4.356 (1.8657 – 10.17) P<0.001 ***
IDH1 Mutation (N=63) 0.252 (0.0809 – 0.78) P=0.017 *
1p19q co-deletion (N=63) 0.086 (0.0097 – 0.77) P=0.028 *
Events: 55; Global p-value (Log-Rank): 8.7188e-11
AIC: 320.77; Concordance Index: 0.78
EZH2-92aa (N=63) 1.1 (1 – 1.1) P=0.001 **
Events: 55; Global p-value (Log-Rank): 0.0059003
AIC: 299.33; Concordance Index: 0.62
EZH2-92aa (N=63) 1.1 (1 – 1.1) P=0.024 *
Events: 55; Global p-value (Log-Rank): 0.041903
AIC: 320.68; Concordance Index: 0.59
EZH2-92aa (N=63) 1.1 (1 – 1.1) P=0.014 *
Events: 55; Global p-value (Log-Rank): 0.028587
AIC: 335.05; Concordance Index: 0.58

Supplementary Figure 2. Murine CircEZH2; identification of the IRES in circEZH2; verification of the unique EZH2-92aa sequence by MS; multivariate analysis of the prognostic value of EZH2-92aa (related to Fig. 2)

a. Comparison of circEZH2 sequences between humans and mice.

b. Comparison of circEZH2-encoded products between humans and mice.

c. Left, sketches of the strategy for circRNA-based IRES verification. MCS, multiple cloning site. Right, WT and different truncated IRESs predicted in circEZH2 RNA were cloned into the circ-Rluc-IRES reporter vector as indicated. 1# vs 3#, *P*=1.34e-05; 3# vs 4#, *P*=6.01e-05; 3# vs 5#, *P*=4.93e-05.

d. The unique C-terminal peptide sequences of human EZH2-92aa were identified by MS.

e. Identification of the unique C-terminal peptide sequence of EZH2-92aa in the mouse glioma cell line GL261.

f. Relative circEZH2 levels and the protein levels of EZH2-92aa in circEZH2 stable KD and control GL261 cells. *P*=4.59e-06.

g. Multivariate Cox regression analysis of the high-grade glioma cohort consisting of 63 patients. Left panel, forest plot of the output results. Right panel, subgroup analysis evaluating the prognostic value of EZH2-92aa after stratification based on age, IDH1 status and 1p19q status. These three variables were stratified into two groups: age (<50 and ≥50 years old), IDH1 status (wild-type and mutant), and 1p19q codeletion (absent and present).

The data are presented as the mean ± SD of three independent experiments. Unpaired two-tailed Student's t test was used to determine the significance of differences between the indicated groups where applicable. **P*<0.05; ***P*<0.01; ****P*<0.001. Source Data are provided as a Source Data file.

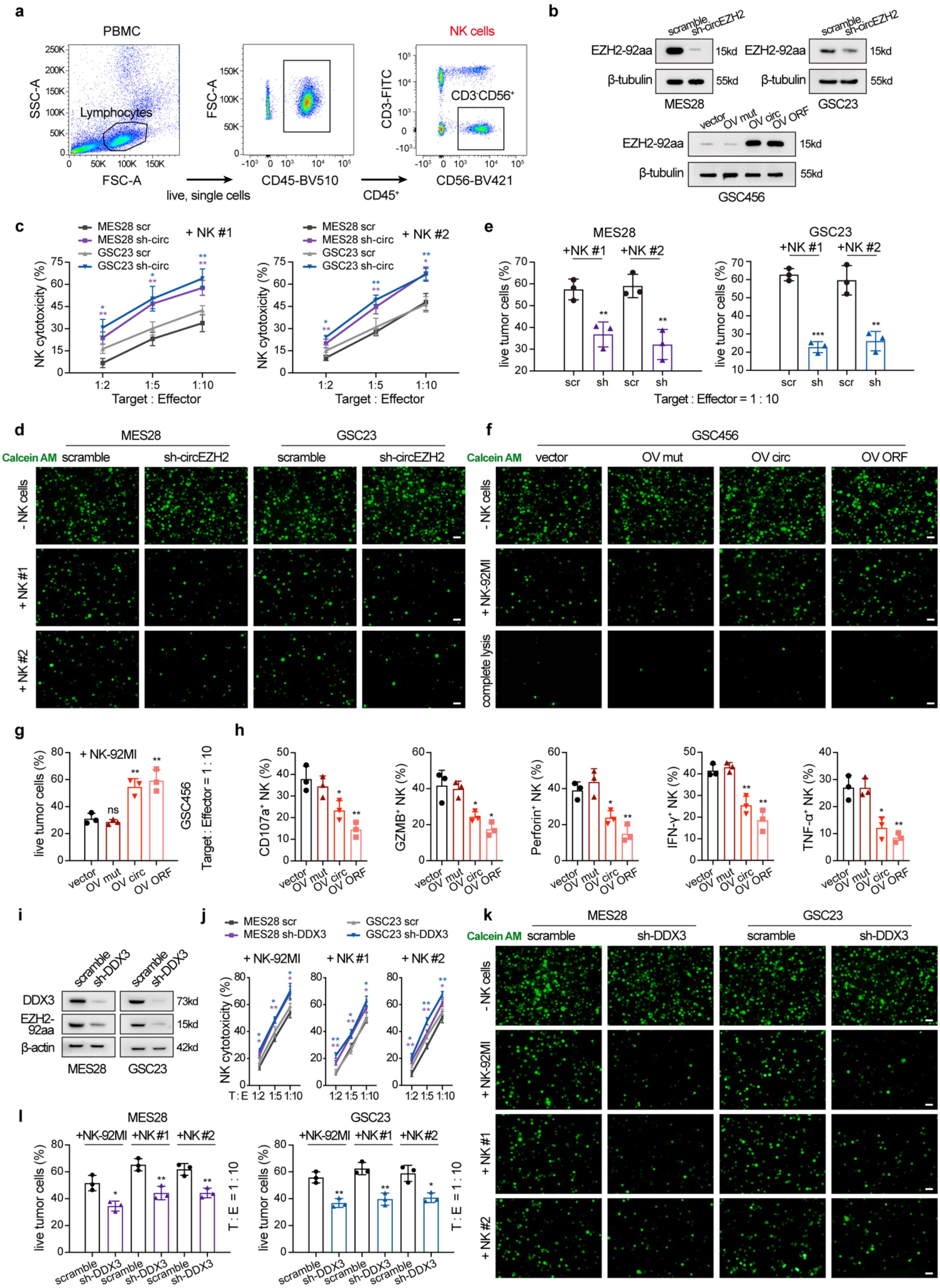

a
PBMC
Lymphocytes
SSC-A
FSC-A
live, single cells
CD45-BV510
CD45⁺
NK cells
CD3-FITC
CD3⁻CD56⁺
CD56-BV421
b
scramble
sh-circEZH2
EZH2-92aa
β-tubulin
15kd
55kd
MES28
GSC23
vector
OV mut
OV circ
OV ORF
GSC456
c
MES28 scr
MES28 sh-circ
GSC23 scr
GSC23 sh-circ
+ NK #1
+ NK #2
NK cytotoxicity (%)
Target : Effector
e
MES28
GSC23
+NK #1
+NK #2
live tumor cells (%)
scr
sh
Target : Effector = 1 : 10
d
Calcein AM
- NK cells
f
+ NK-92MI
complete lysis
g
+ NK-92MI
GSC456
Target : Effector = 1 : 10
h
CD107a⁺ NK (%)
GZMB⁺ NK (%)
Perforin⁺ NK (%)
IFN-γ⁺ NK (%)
TNF-α⁺ NK (%)
i
scramble
sh-DDX3
DDX3
EZH2-92aa
β-actin
73kd
15kd
42kd
j
MES28 sh-DDX3
GSC23 sh-DDX3
T : E
k
l
+NK-92MI
T : E = 1 : 10

Supplementary Figure 3. EZH2-92aa abrogates NK cell cytotoxicity (related to Fig. 4)

a. Gating strategy for identifying and sorting primary NK cells in human peripheral blood. PBMCs, peripheral blood mononuclear cells.

b. Top, Protein levels of EZH2-92aa in circEZH2 stable KD MES28/GSC23 and control cells. Bottom, Protein levels of EZH2-92aa in GSC456 cells stably overexpressed with vector, mutated circEZH2 with deletion of ATG (OV mut), circEZH2 (OV circ) and circEZH2 ORF (OV ORF).

c. GSCs with stable circEZH2 KD or control were cocultured with two cultures of primary NK cells from donors #1 and #2 at different T:E ratios for 2 hours. LDH activity in the supernatant was measured to calculate NK cell cytotoxicity. scr, scrambled shRNA control; sh, sh-circEZH2 RNA. NK #1, scr vs sh-circ, MES28, 1:2 *P*=0.0046, 1:5 *P*=0.0018, 1:10 *P*=0.0058, GSC23, 1:2 *P*=0.0175, 1:5 *P*=0.0210, 1:10 *P*=0.0064; NK #2, scr vs sh-circ, MES28, 1:2 *P*=0.0083, 1:5 *P*=0.0055, 1:10 *P*=0.0102, GSC23, 1:2 *P*=0.0161, 1:5 *P*=0.0092, 1:10 *P*=0.0072.

d. GSCs (1×10^5 cells/ml) were stained with calcein AM and seeded with two cultures of primary NK cells from donors #1 and #2 (1×10^6 cells/ml) at a T:E ratio of 1:10. Images of fluorescent cells (live cells) were acquired after an incubation for 2 hours. Scale bar, 50 μm.

e. Quantification of the remaining live cells in **(d)**. scr vs sh, NK #1, MES28 *P*=0.0086, GSC23 *P*=0.0010; NK #2, MES28 *P*=0.0059, GSC23 *P*=0.0039.

f. GSC456 cells overexpressed with indicated constructs (1×10^5 cells/ml) were stained with calcein AM and seeded with NK cells (1×10^6 cells/ml) at a T:E ratio of 1:10. Images of fluorescent cells (live cells) were acquired after an incubation for 2 hours. Scale bar, 50 μm.

g. Quantification of remaining live cells in **(f)**. vector vs OV circ, *P*=0.0051; vector vs OV ORF, *P*=0.0059.

h. Quantification of the expression of the indicated molecules in NK cells after incubation with GSC456 cells overexpressed with indicated constructs. vector vs OV circ, CD107a *P*=0.0286, GZMB *P*=0.0279, Perforin *P*=0.0125, IFN-γ *P*=0.0056, TNF-α *P*=0.0124; vector vs OV ORF, CD107a *P*=0.0042, GZMB *P*=0.0104, Perforin *P*=0.0034, IFN-γ *P*=0.0023, TNF-α *P*=0.0027.

i. Protein levels of EZH2-92aa in DDX3 stable KD MES28/GSC23 and control cells.

j. GSCs with stable DDX3 KD or control were cocultured with NK-92MI and primary NK cells at

different T:E ratios for 2 hours. LDH activity in the supernatant was measured to calculate NK cell cytotoxicity. scr vs sh-DDX3, NK-92MI, MES28, 1:2 *P*=0.0116, 1:5 *P*=0.0019, 1:10 *P*=0.0190, GSC23, 1:2 *P*=0.0150, 1:5 *P*=0.0275, 1:10 *P*=0.0306; NK #1, MES28, 1:2 *P*=0.0030, 1:5 *P*=0.0090, 1:10 *P*=0.0175, GSC23, 1:2 *P*=0.0015 ,1:5 *P*=0.0117, 1:10 *P*=0.0143; NK #2, MES28, 1:2 *P*=0.0066, 1:5 *P*=0.0033, 1:10 *P*=0.0231, GSC23, 1:2 *P*=0.0103, 1:5 *P*=0.0044, 1:10 *P*=0.0020.

k. GSCs (1×10^5 cells/ml) with stable DDX3 stable KD or control were stained with calcein AM and seeded with NK cells (1×10^6 cells/ml) at a T:E ratio of 1:10. Images of fluorescent cells (live cells) were acquired after an incubation for 2 hours. Scale bar, 50 μm.

l. Quantification of the remaining live cells in **(j)**. scr vs sh-DDX3, NK-92MI, MES28 *P*=0.0120, GSC23 *P*=0.0035; NK #1, MES28 *P*=0.0057, GSC23 *P*=0.0036; NK #2, MES28 *P*=0.0058, GSC23 *P*=0.0111.

The data are presented as the mean ± SD of three independent experiments. Two-tailed unpaired t test. **P*<0.05; ***P*<0.01; ****P*<0.001. Source Data are provided as a Source Data file.

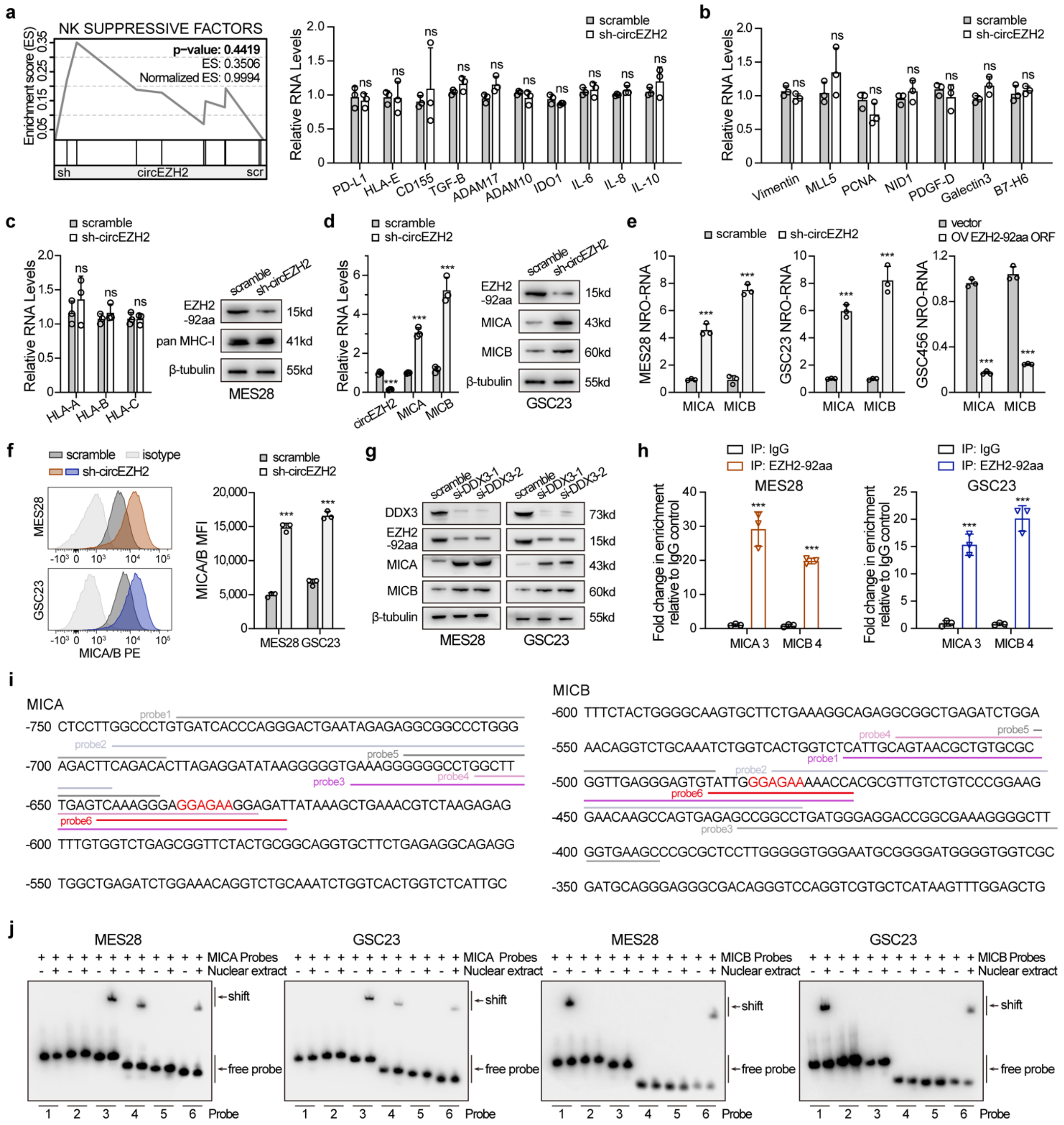

Supplementary Figure 4. CircEZH2 expression is not significantly correlated with NK dysfunction-related factors, NCR ligands and MHC-I; EZH2-92aa directly represses the transcription of NKG2D ligands; putative binding sites of EZH2-92aa (related to Fig. 5)

a. Left, GSEA of an established set of NK dysfunction markers in circEZH2 stable KD and control MES28 GSCs. Right, RNA levels of NK dysfunction factors in circEZH2 stable KD and control MES28 GSCs.

b. Relative RNA levels of NCR ligands in circEZH2 stable KD and control MES28 GSCs.

c. Relative MHC-I RNA and protein levels in circEZH2 stable KD and control MES28 GSCs.

d. Left panel, relative MICA/MICB RNA levels in circEZH2 stable KD GSC23 GSCs. Right, immunoblot showing the levels of MICA/MICB in circEZH2 stable KD GSC23 GSCs. scramble vs sh-circEZH2, circEZH2 *P*=2.83e-05, MICA *P*=1.37e-04, MICB *P*=5.11e-04.

e. NRO-RT–qPCR assays showed upregulated or downregulated MICA/MICB nascent transcription in circEZH2 stable KD or EZH2-92aa stable OV GSCs. MES28, MICA *P*=1.67e-04, MICB *P*=2.50e-05; GSC23, MICA *P*=5.30e-05, MICB *P*=2.60e-04; GSC456, MICA *P*=3.56e-06, MICB *P*=3.01e-05.

f. Representative histogram (left panel) and quantification (right panel) of the mean fluorescence intensity (MFI) of MICA/B in circEZH2 stable KD or control GSCs using an anti-MICA/B-PE-conjugated antibody. MES28, *P*=1.51e-05; GSC23, *P*=2.52e-05.

g. MICA/MICB protein levels in DDX3 knockdown GSCs.

h. ChIP-qPCR analysis of the EZH2-92aa binding site in the MICA/MICB promoters in MES28 and GSC23 GSCs using the custom anti-EZH2-92aa antibody. IgG vs EZH2-92aa, MES28, MICA3 *P*=6.62e-04, MICB4 *P*=3.45e-06; GSC23, MICA3 *P*=2.42e-04, MICB4 *P*=1.41e-04.

i. Putative binding sites of EZH2-92aa in the MICA/MICB promoters and the design of corresponding EMSA probes.

j. EMSA was performed using the nuclear extracts of MES28 and GSC23 GSCs and six specific biotin-labelled probes.

The data are presented as the mean ± SD of three independent experiments. Unpaired two-tailed Student’s t test was used to determine the significance of differences between the indicated groups where applicable. *P<0.05; **P<0.01; ***P<0.001. Source Data are provided as a Source Data file.

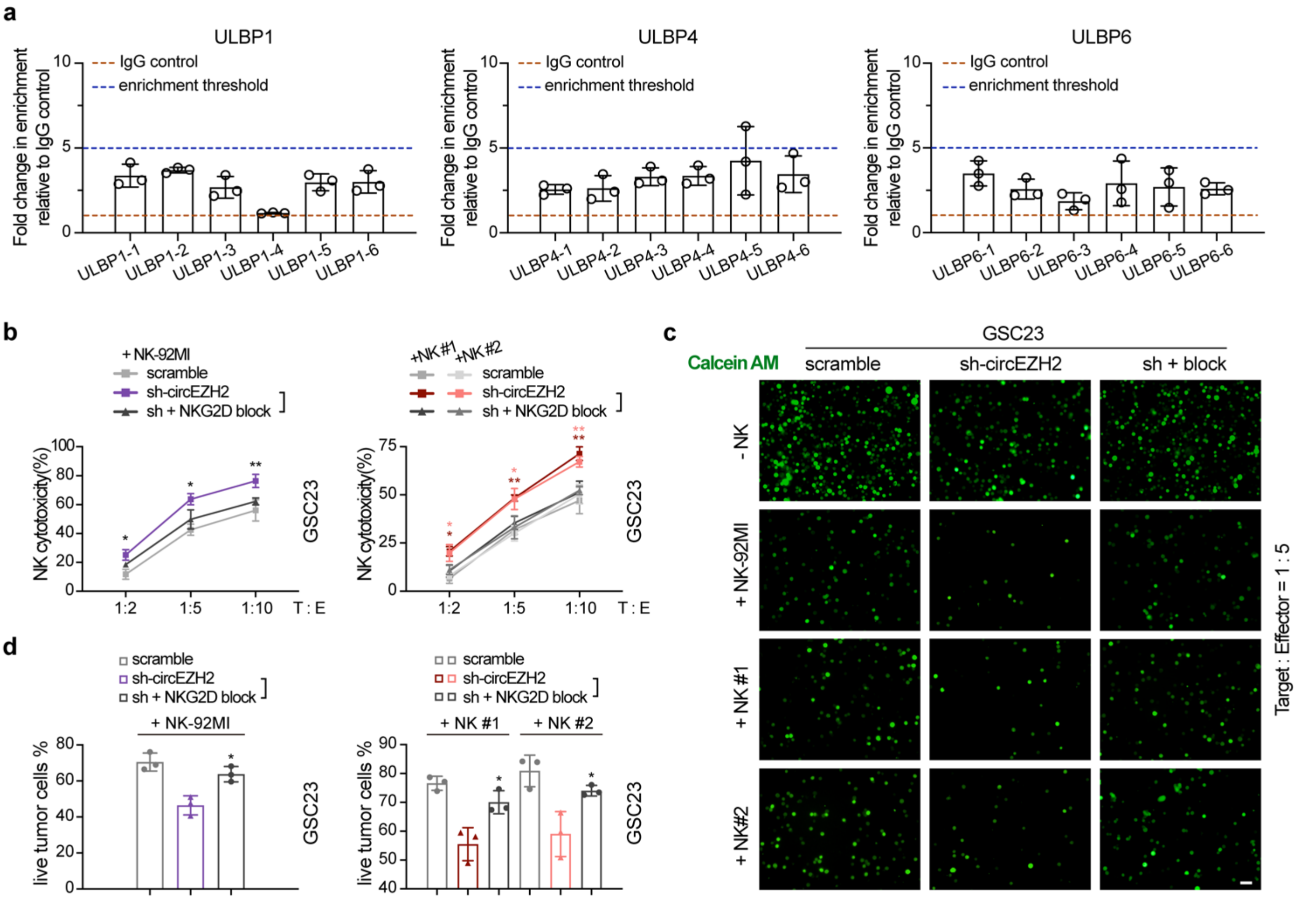

Supplementary Figure 5. EZH-92aa indirectly represses ULBPs; EZH2-92aa inhibits NK cytotoxicity via the NKG2DL-NKG2D axis (related to Fig. 6)

a. ChIP-qPCR was performed to analyse the recruitment of EZH2-92aa to the promoters of ULBP1/4/6 in GSCs overexpressing EZH2-92aa-3×flag.

b. GSC23 cells with stable circEZH2 KD, stable circEZH2 KD plus NKG2D blockade (10 μg/ml) or control GSCs were cocultured with NK cells (NK-92MI cells and two cultures of primary NK cells from donors #1 and #2) at different T:E ratios for 2 hours. LDH activity in the supernatant was measured to calculate NK cell cytotoxicity. Sh-circEZH2 vs sh-circEZH2 plus block, NK-92MI, 1:2 P=0.0483, 1:5 P=0.0347, 1:10 P=0.0091; NK #1, 1:2 P=0.0116, 1:5 P=0.0043, 1:10 P=0.0071; NK #2, 1:2 P=0.0370, 1:5 P=0.0343, 1:10 P=0.0018.

c. GSC23 cells (1×10^5 cells/ml) with the indicated modifications were stained with calcein AM and seeded with NK cells (1×10^6 cells/ml) at a T:E ratio of 1:5. NKG2D block (10 μg/ml) were added in the indicated groups. Images of fluorescent cells (live cells) were acquired after an incubation

for 2 hours. Scale bar, 50 μm.

d. Quantification of the remaining live GSCs in **(c)**. Sh-circEZH2 vs sh-circEZH2 plus block, NK-92MI, *P*=0.0117; NK #1, *P*=0.0228; NK #2, *P*=0.0310.

The data are presented as the mean ± SD of three independent experiments. Unpaired two-tailed Student's t test was used to determine the significance of the differences between the indicated groups. ns, nonsignificant, **P*<0.05, ***P*<0.01, and ****P*<0.001. Source Data are provided as a Source Data file.

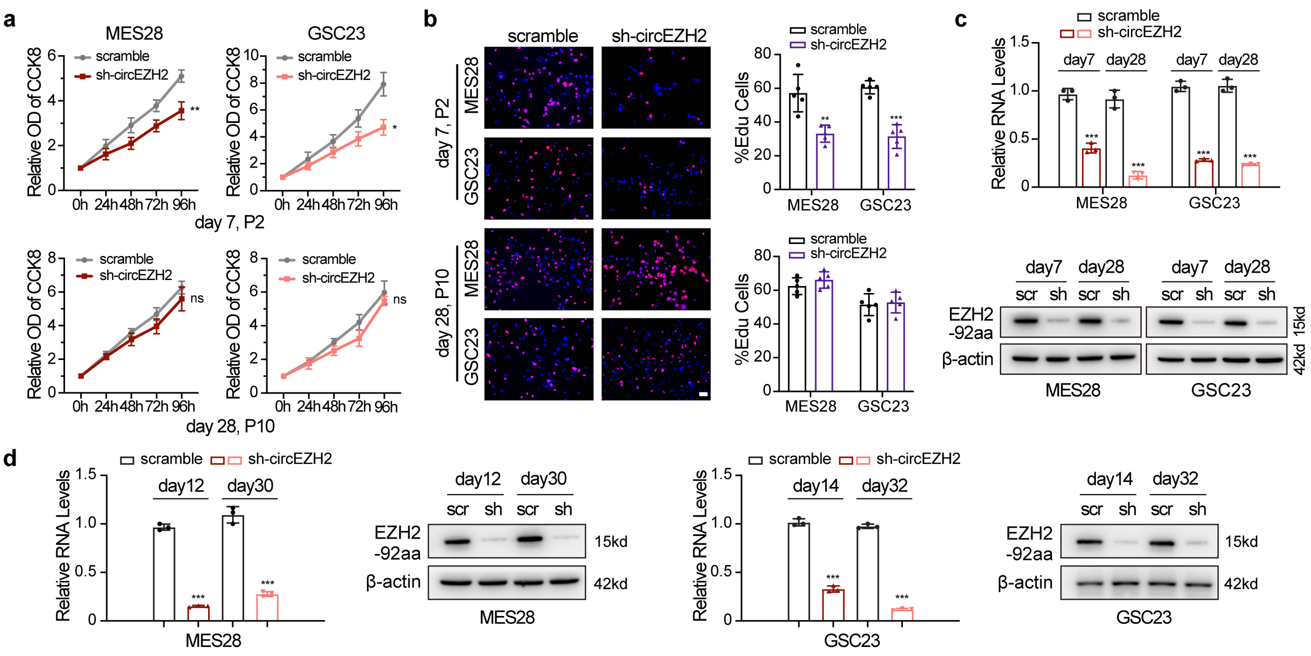

Supplementary Figure 6. Short-term inhibitory effect of EZH2-92aa KD on the growth of GSCs *in vitro*; validation of EZH-92aa KD in GSCs from late-passage GSCs and late-stage xenografts (related to Fig. 7)

a. Proliferation of control and circEZH2 stable KD MES28 and GSC23 cells. P = cell passage number. Day 7 P2, MES28, *P*=0.0023; GSC23, *P*=0.0111.

b. Left, EdU incorporation assay of control and circEZH2 stable KD MES28 and GSC23 cells. Scale bar, 50 μm. Right, quantification of EdU-positive cells in the indicated groups. Day 7 P2, MES28, *P*=0.0022; GSC23, *P*=4.10e-05.

c. Relative RNA levels of circEZH2 (top panel) and EZH2-92aa protein levels (bottom panel) in early- and late-passage control GSCs or GSCs with stable circEZH2 KD. Day 7 P2, MES28, *P*=2.26e-04; GSC23, *P*=1.72e-05; day 28 P10, MES28, *P*=1.65e-04; GSC23, *P*=3.04e-05.

d. Relative RNA levels of circEZH2 (left panels) and EZH2-92aa protein levels (right panels) in GSCs from primary cultures of xenografts isolated from early- or late-stage GBM mouse models. MES28, day12 *P*=1.75e-06, day 30 *P*=8.79e-05; GSC23, day14 *P*=1.60e-05, day32 *P*=4.62e-07.

The data are pooled from at least three independent experiments. The data are presented as the means ± SD. Two-way ANOVA in **(a)** and unpaired two-tailed Student's t test were used to determine the significance of differences between the indicated groups where applicable. **P*<0.05, ***P*<0.01, and ****P*<0.001. Source Data are provided as a Source Data file.

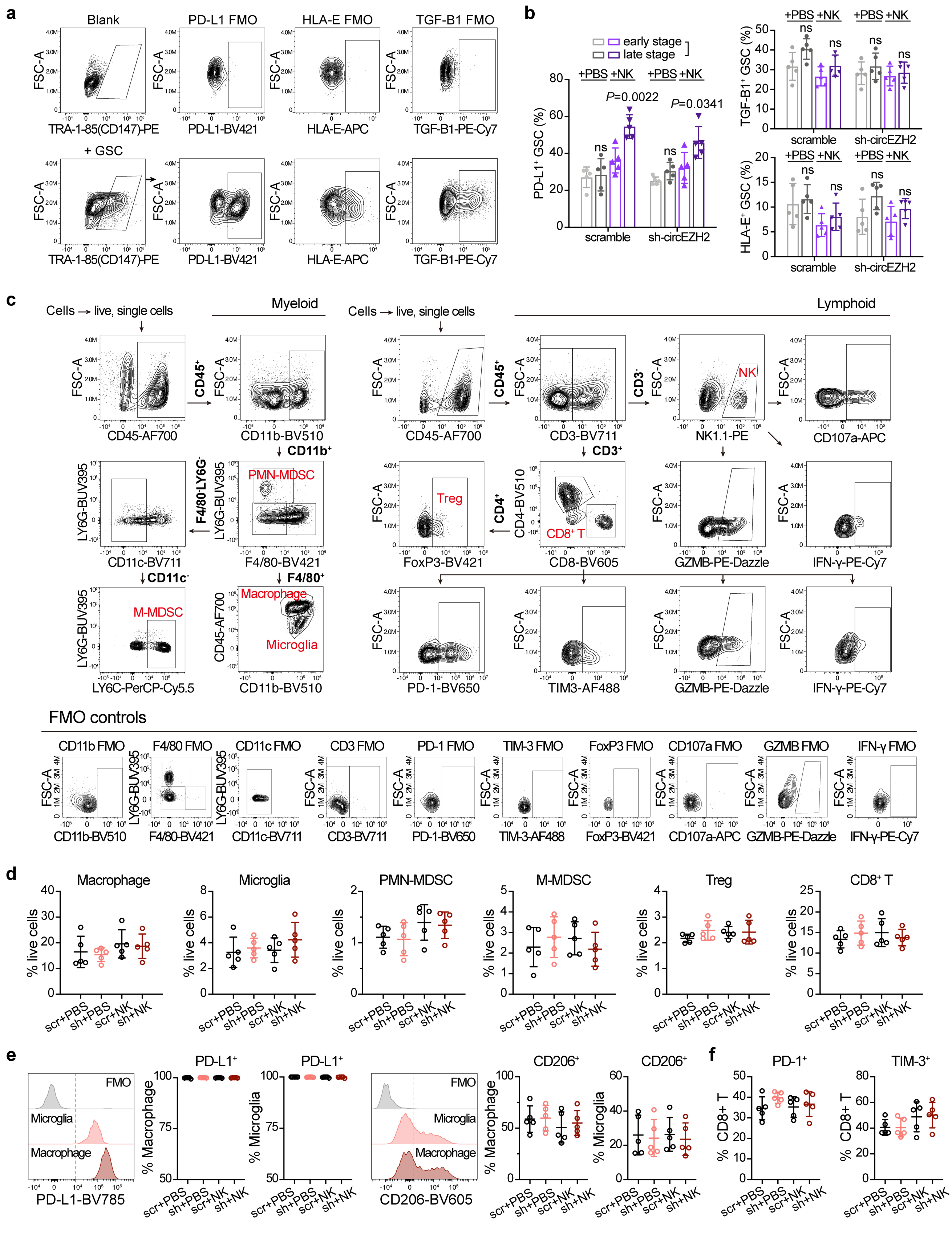
a
Blank
PD-L1 FMO
HLA-E FMO
TGF-B1 FMO
+ GSC
TRA-1-85(CD147)-PE
PD-L1-BV421
HLA-E-APC
TGF-B1-PE-Cy7
b
early stage
late stage
+PBS +NK
P=0.0022
P=0.0341
PD-L1+ GSC (%)
TGF-B1+ GSC (%)
HLA-E+ GSC (%)
scramble
sh-circEZH2
c
Cells → live, single cells
Myeloid
Lymphoid
CD45+
CD11b+
F4/80-LY6G-
CD11c-
F4/80+
PMN-MDSC
M-MDSC
Macrophage
Microglia
CD3-
CD3+
CD4+
NK
Treg
CD8+ T
FMO controls
d
Macrophage
Microglia
PMN-MDSC
M-MDSC
Treg
CD8+ T
% live cells
scr+PBS
sh+PBS
scr+NK
sh+NK
e
PD-L1+
CD206+
FMO
% Macrophage
% Microglia
PD-L1-BV785
CD206-BV605
f
PD-1+
TIM-3+
% CD8+ T

Supplementary Figure 7. Alternative suppressive factors in late-stage xenografts (related to Fig. 7)

a. Left panel, TRA-1-85 (human-specific antigen) was used to identify human GSCs from the implanted xenografts. Right panel, representative contour chart to identify positive cells expressing the indicated molecules. The FMO control represents a fully stained sample minus the indicated antibody.

b. Quantification of the frequencies of PD-L1$^+$, TGF-B1$^+$ and HLA-E$^+$ GSCs isolated from the early- or late-stage tumour mass of the NDG GBM model (n=5 per group).

c. Gating strategy used to define myeloid and lymphoid subsets in the C57BL/6 GBM model. The FMO control represents a fully stained sample minus the indicated antibody.

d. Quantification of indicated immune cell subsets from the late stage tumour mass of the C57BL/6 GBM model (n=5 samples per group). No significant differences in frequency of the indicated subsets were observed between the groups.

e. Representatively histogram and quantification of CD206$^+$ and PD-L1$^+$ macrophages and microglia from the late stage tumour mass of the C57BL/6 GBM model (n=5 samples per group). No significant differences in frequency of the indicated cells were observed between the groups.

f. Quantification of PD-1$^+$ and TIM-3$^+$ CD8$^+$ T cells from the late stage tumour mass of the C57BL/6 GBM model (n=5 samples per group). No significant differences in frequency of the indicated cells were observed between the groups.

The data are presented as the means ± SD. Unpaired two-tailed Student's t test were used to determine the significance of differences between the indicated groups where applicable. ns, non-significant, **P*<0.05, ***P*<0.01, and ****P*<0.001. Source Data are provided as a Source Data file.

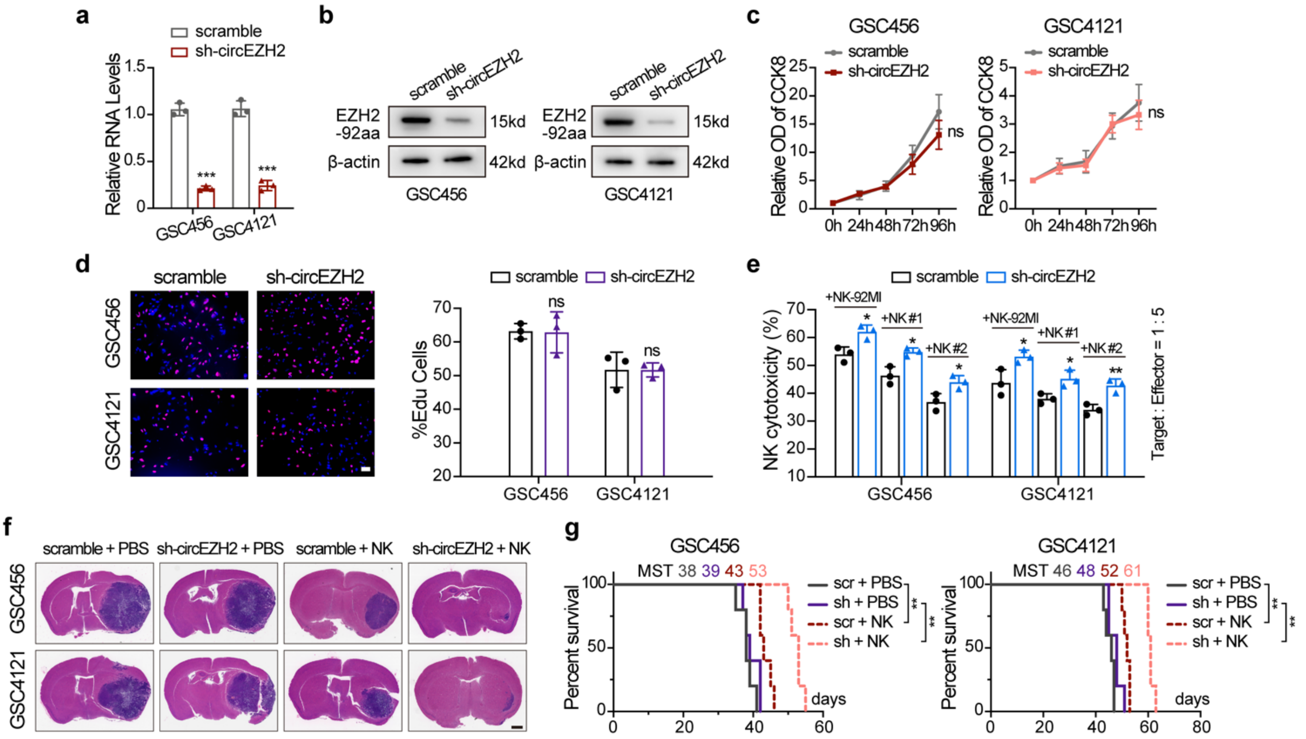

Supplementary Figure 8. Effect of EZH2-92aa KD on the proliferation and NK cytotoxicity sensitivity of GSC456/4121

a. Relative RNA levels of circEZH2 in GSC456/GSC4121 cells with stable circEZH2 KD. GSC456, *P*=3.34e-05; GSC4121, *P*=1.39e-04.

b. EZH2-92aa protein levels in GSC456/GSC4121 cells with stable circEZH2 KD.

c. Proliferation of control and circEZH2 stable KD GSC456 and GSC4121 cells.

d. Left panel, EdU incorporation assay of control and circEZH2 stable KD GSC456 and GSC4121 cells. Scale bar, 50 μm. Right panel, quantification of EdU-positive cells in the indicated groups.

e. LDH activity in the supernatant was measured to calculate the cytotoxicity of NK cells cocultured with indicated GSC cells at a target:effector ratio of 1:5. GSC456, NK-92MI *P*=0.0178, NK #1 *P*=0.0141, NK #2 *P*=0.0318; GSC4121, NK-92MI *P*=0.0359, NK #1 *P*=0.0255, NK #2 *P*=0.0080.

f. Representative images of H&E-stained brain slices from mice intracranially implanted with circEZH2 stable KD GSC456 and GSC4121 cells and the corresponding scrambled control cells after treatment with PBS or NK cells (n = 5 mice per group). Scale bar, 1 mm.

g. Kaplan-Meier analysis of mice intracranially implanted with GSC456 and GSC4121 cells with stable KD of circEZH2 and the corresponding scrambled control cells and treated with PBS or NK

cells (n = 5 mice per group). Log-rank test. MST, median survival time. Scramble+NK vs scramble, GSC456 *P*=0.0018, GSC4121 *P*=0.0027; sh-circEZH2+NK vs sh-circEZH2, GSC456 *P*=0.0027, GSC4121 *P*=0.0019.

The data in **(a)-(e)** are pooled from three independent experiments. The data are presented as the means ± SD. Unpaired two-tailed Student's t test was used to determine the significance of differences between the indicated groups where applicable. ns, non-significant, **P*<0.05, ***P*<0.01, and ****P*<0.001. Source Data are provided as a Source Data file.

Supplementary Table 1 NK cell activation gene set[1]

**Cytokines**	*IFNG, TNF, LTA, CSF1, CSF2, CCL3, CCL4, CCL4L2, XCL1, XCL2*
**Transcription factors**	*EGR2, EGR3, IRF4, EOMES*
**Surface markers**	*TNFSF4, TNFRSF9*
**Effector markers**	*GZMB, LAMP1, PRF1, KLRK1, TNRSF7*
**Others**	*CCL3L1, CCL3L3, FOXO1, NFIL3, PRDM1, NCR1, NCR2, NCR3*

Supplementary Table 2 NK cell-activating factors

**SYMBOL**	**ENSEMBL ID**
MICA	ENSG00000204520
MICB	ENSG00000204516
ULBP1	ENSG00000111981
ULBP2	ENSG00000131015
ULBP3	ENSG00000131019
ULBP4	ENSG00000164520
ULBP5	ENSG00000203722
ULBP6	ENSG00000155918
CD48	ENSG00000117091
ICAM1	ENSG00000090339
B7-H6	ENSG00000188211
B7-H7	ENSG00000114455
CD58	ENSG00000116815
IL-2	ENSG00000109471
IL-15	ENSG00000164136
IL-18	ENSG00000150782
IL-12A	ENSG00000168811
IL-12B	ENSG00000113302
IL-21	ENSG00000138684

Supplementary Table 3 RNA oligos

siRNAs	sense 5'-3'	antisense 5'-3'
scramble siRNA	CAGAAUACUCCUCGUGAGAtt	UCUCACGAGGAGUAUUCUGtt
circEZH2 siRNA1	GACUAGGGAGAAUAAUCAUGGTT	CCAUGAUUAUUCUCCCUAGUCTT
circEZH2 siRNA2	GGACUAGGGAGAAUAAUCAUGTT	CAUGAUUAUUCUCCCUAGUCCTT
DDX3 siRNA1	CCUGAACUCUUCAGAUAAUTT	AUUAUCUGAAGAGUUCAGGTT
DDX3 siRNA2	GCUGGCUCGUGAUUUCUUATT	UAAGAAAUCACGAGCCAGCTT
DDX60L siRNA1	GGUUUCAACUGCAAUACAUTT	AUGUAUUGCAGUUGAAACCTT
DDX60L siRNA2	GGAAUUGUGUCCAGAGGAATT	UUCCUCUGGACACAAUUCCTT

shRNAs	
circEZH2 shRNA1	GACTAGGGAGAATAATCATGG
circEZH2 shRNA2	GGACTAGGGAGAATAATCATG
DDX3 shRNA	CCTGAACTCTTCAGATAAT

FISH probes	
circEZH2	5' CY3-TGGCCCATGATTATTCTCCCTAGTCCCGCGC 3'

Supplementary Table 4 PCR Primers

RIP primers	
circEZH2 IRES F	TGAGAAGGGACCAGTTTGTT
circEZH2 IRES R	AGTCTGGCCCATGATTATTCTCC
BCL2 F	CCCGCGACTCCTGATTCATT
BCL2 R	CAGTCTACTTCCTCTGTGATGTTGT

ChIP primers	
MICA 1 F	GCGTTGAAGGAGCTGACTTT
MICA 1 R	CGTCTGCCACCATATCATCC
MICA 2 F	CACATTGGAGGGGACTATGG
MICA 2 R	GTGATCACAGGGCCAAGG
MICA 3 F	TGATCACCCAGGGACTGAAT
MICA 3 R	TCTCCTTCTCCTCCCTTTGA
MICA 4 F	TGAAACGTCTAAGAGAGTTTGTGG
MICA 4 R	CGTTACTGGCAATGAGACCA
MICA 5 F	TCATTGCCAGTAACGCTGTG

MICA 5 R	CCTCACCAAGCCCCTTTC
MICA 6 F	GAAAGGGGCTTGGTGAGG
MICA 6 R	GCTGAGAGTACAGCTCCAACTTC
MICA 7 F	GCCGTGCTTATGAAGTTGGA
MICA 7 R	CTGGAGACCTGGGGAGATTT
MICA 8 F	CCCCAGTTTCATTGGATGAG
MICA 8 R	CAGCCAGAAGCAGAAAGACC
MICB 1 F	TGTCATATTGAAGGGGACTATGG
MICB 1 R	GTAATCACAGGGCCAACGAG
MICB 2 F	TTGGCCCTGTGATTACCC
MICB 2 R	ATCTCCTTCTCCTCCCTTCG
MICB 3 F	GCTGAAACGTCTAAGAGAATTTG
MICB 3 R	GCGTTACTGCAATGAGACCA
MICB 4 F	TCATTGCAGTAACGCTGTGC
MICB 4 R	GCTTCACCAAGCCCCTTT
MICB 5 F	GAAAGGGGCTTGGTGAAG
MICB 5 R	GCCCCGAGTAGCTGAGAGTA
MICB 6 F	GGTCCAGGTCGTGCTCATAA
MICB 6 R	AATTCTAGCGGCCTGGAGAC
MICB 7 F	CAGGCCGCTAGAATTTTCTCT
MICB 7 R	CCCGTCGAAATTTAGTCACC
MICB 8 F	ATTCAGTTGGCCACTGCTG
MICB 8 R	CAGGAACCCCACTCACCA
ULBP1-1 F	CTCTGCCCAGTCCTTACTGC
ULBP1-1 R	AGCCATCCTCAAAAGGGACG
ULBP1-2 F	CTGAGCCGAGGTGCAGATAC
ULBP1-2 R	AGCCGAGGGAAGAGTCTACG
ULBP1-3 F	TGATTGATCACGGTAGCCCTC
ULBP1-3 R	CCATTCCGGAGCGAACCTTT
ULBP1-4 F	TTCGCTCCGGAATGGGGAA
ULBP1-4 R	GAATACTCAGTGGCGGCGG
ULBP1-5 F	GCCGCCACTGAGTATTCCTA
ULBP1-5 R	CGTTCCTTCCGTTCCTTCCA
ULBP1-6 F	CCATGGAAGGAACGGAAGGA
ULBP1-6 R	GGGATCCTAGGCATGCACTTT
ULBP4-1 F	GCCTGGCCATTTCAGTTTCGTT
ULBP4-1 R	GTGGTCTACCATGACAGATGGG
ULBP4-2 F	ATGGTAGACCACTTGCTGCC
ULBP4-2 R	CCACTGTCCCTAACCCGTTC
ULBP4-3 F	GAACGGGTTAGGGACAGTGG
ULBP4-3 R	CTGGTGTGAGCAGGATGGTT

ULBP4-4 F	TCCAACCATCCTGCTCACAC
ULBP4-4 R	GTCAGGCCTGTAGAGACCCA
ULBP4-5 F	CCTCAGTTGTTCCAGGGTAAAGA
ULBP4-5 R	CATTCACCCTCACTGGTATGG
ULBP4-6 F	CCTGACTTCTAGCCCTGTGC
ULBP4-6 R	TCCCTCCTCCTTGTCCTCAG
ULBP6-1 F	AGCGTGTCACAGGTTGAGAT
ULBP6-1 R	TCTTGCTTCTGCTTCACCCA
ULBP6-2 F	TGCACTGGGTGAAGCAGAAG
ULBP6-2 R	GCAGGTAGGGTTTCAGAGGTT
ULBP6-3 F	GGGACTGGGGAAGTAGTGGA
ULBP6-3 R	AGGAATATTCAGCGGCCTCG
ULBP6-4 F	TCTGTCGTGGAAGGAATCGTG
ULBP6-4 R	GTTACTGGGGAATGTACCGGG
ULBP6-5 F	CAGCCTTTCAGCACAGAGGT
ULBP6-5 R	TCCTTCCAGAAGCCTTCCCT
ULBP6-6 F	AACCTGAGGGACTGTGGACT
ULBP6-6 R	CCTCGTTTTCACCCTGCTCT

**RT-qPCR primers**	
circEZH2 F	GGTTCAGACGAGCTGATGAAGT
circEZH2 R	AGTCTGGCCCATGATTATTCTCC
EZH2 F	CCATCCAGACTGGCGAAGAG
EZH2 R	CTGAAGCTAAGGCAGCTGTT
circHIPK3 F	TCGGCCAGTCATGTATCAAA
circHIPK3 R	ACCAAGACTTGTGAGGCCAT
U6 F	CTCGCTTCGGCAGCACA
U6 R	AACGCTTCACGAATTTGCGT
b-actin F	CATGTACGTTGCTATCCAGGC
b-actin R	CTCCTTAATGTCACGCACGAT
b-actin F (for NRO)	AGCTCATTGTAGAAGGTGTGG
b-actin R (for NRO)	GGCATGGGTCAGAAGGATTC
ULBP1 F	TAAGTCCAGACCTGAACCACA
ULBP1 R	TCCACCACGTCTCTTAGTGTT
ULBP2 F	AGCAACTGCGTGACATTCAG
ULBP2 R	GCCATCCTATACAGTCTCCCA
ULBP3 F	AGCTTCGATGGACGGAAGTTC
ULBP3 R	TCAGTCCGCTATCCTTCTCCC
ULBP4 F	CCTCCTCTTTGACGCAATGAA
ULBP4 R	CCTCCCAGTGCCCTAAGAA
ULBP5 F	GACAGCTACCAAATAGCGAAGC

ULBP5 R	GGTAAGGAGTGTGAGTCGTCT
ULBP6 F	ACATCACCGTCATCCCTAAGT
ULBP6 R	GTCTTGTTGCCACAGTCATAGT
MICA F	AGGGTTTCTTGCTGAGGTACA
MICA R	GGTCTCTCTGTCCCATGTCTTA
MICB F	TGGAGACTCAAGAATCGACAGT
MICB R	CTGCATAGCGCGATAGTGTG
CD274(PD-L1) F	GGACAAGCAGTGACCATCAAG
CD274(PD-L1) R	CCCAGAATTACCAAGTGAGTCCT
HLA-E F	TTCCGAGTGAATCTGCGGAC
HLA-E R	GTCGTAGGCGAACTGTTCATAC
CD155 F	GGACGGCAAGAATGTGACCT
CD155 R	GGTCGTGCTCCAATTATAGCCT
TGF-B F	CTAATGGTGGAAACCCACAACG
TGF-B R	TATCGCCAGGAATTGTTGCTG
ADAM17 F	TTTCACGTTTGCAGTCTCCAA
ADAM17 R	AGAAGCGATGATCTGCTACCA
ADAM10 F	TTTCAACCTACGAATGAAGAGGG
ADAM10 R	TAAAATGTGCCACCACGAGTC
IDO1 F	TCTCATTTCGTGATGGAGACTGC
IDO1 R	GTGTCCCGTTCTTGCATTTGC
IL-6 F	ACTCACCTCTTCAGAACGAATTG
IL-6 R	CCATCTTTGGAAGGTTCAGGTTG
IL-8 F	ACTGAGAGTGATTGAGAGTGGAC
IL-8 R	AACCCTCTGCACCCAGTTTTC
IL10-2 F	GACTTTAAGGGTTACCTGGGTTG
IL10-2 R	TCACATGCGCCTTGATGTCTG
B7-H6 F	TGTGAGTCAAGTGGGTTCTACC
B7-H6 R	CATGCCGTACCACACACTG
Galectin3 F	ATGGCAGACAATTTTTCGCTCC
Galectin3 R	GCCTGTCCAGGATAAGCCC
MLL5 F	AAATCCAACAGTTATCCCCACC
MLL5 R	CGCATAGGGCAAACCAATGTA
PCNA F	ACACTAAGGGCCGAAGATAACG
PCNA R	ACAGCATCTCCAATATGGCTGA
NID1 F	TCTACGTCACCACAAATGGCA
NID1 R	GCGACTGCACCGAATGTTG
PDGF-D F	TCAAGTCCGATGACTACTTTGTG
PDGF-D R	GTGACAGATTCCCAGTTGGTC
Vimentin F	AGTCCACTGAGTACCGGAGAC
Vimentin R	CATTTCACGCATCTGGCGTTC

HLA-A F	GCTCCCACTCCATGAGGTAT
HLA-A R	AGTCTGTGACTGGGCCTTCA
HLA-B F	ACTGAGCTTGTGGAGACCAGA
HLA-B R	GCAGCCCCTCATGCTGT
HLA-C F	CTGGCCCTGACCGAGACCTG
HLA-C R	CGCTTGTACTTCTGTGTCTCC

Supplementary Table 5 EMSA Probes

**EMSA probes**		
MICA probe 1	F	TGATCACCCAGGGACTGAATAGAGAGGCGGCCCTGGGAGACTTCAGACA
MICA probe 1	R	TGTCTGAAGTCTCCCAGGGCCGCCTCTCTATTCAGTCCCTGGGTGATCA
MICA probe 2	F	CAGACACTTAGAGGATATAAGGGGGTGAAAGGGGGGCCTGGCTTTGAGTC
MICA probe 2	R	GACTCAAAGCCAGGCCCCCCTTTCACCCCCTTATATCCTCTAAGTGTCTG
MICA probe 3	F	TGAAAGGGGGGCCTGGCTTTGAGTCAAAGGGAGGAGAAGGAGA
MICA probe 3	R	TCTCCTTCTCCTCCCTTTGACTCAAAGCCAGGCCCCCCTTTCA
MICA probe 4	F	TGGCTTTGAGTCAAAGGGAGGAGAAGG
MICA probe 4	R	CCTTCTCCTCCCTTTGACTCAAAGCCA
MICA probe 5	F	GGGGGCCTGGCTTTGAGTCAAAGG
MICA probe 5	R	CCTTTGACTCAAAGCCAGGCCCCC
MICA probe 6	F	TCAAAGGGAGGAGAAGGAGA
MICA probe 6	R	TCTCCTTCTCCTCCCTTTGA
MICB probe 1	F	TCATTGCAGTAACGCTGTGCGCGGTTGAGGGAGTGTATTGGGAGAAAAACC
MICB probe 1	R	GGTTTTTCTCCCAATACACTCCCTCAACCGCGCACAGCGTTACTGCAATGA
MICB probe 2	F	AGAAAAACCACGCGTTGTCTGTCCCGGAAGGAACAAGCCAGTGAGAGCCGGCC
MICB probe 2	R	GGCCGGCTCTCACTGGCTTGTTCCTTCCGGGACAGACAACGCGTGGTTTTTCT
MICB probe 3	F	GCCGGCCTGATGGGAGGACCGGCGAAAGGGGCTTGGTGAAGC
MICB probe 3	R	GCTTCACCAAGCCCCTTTCGCCGGTCCTCCCATCAGGCCGGC
MICB probe 4	F	CAGTAACGCTGTGCGC
MICB probe 4	R	GCGCACAGCGTTACTG
MICB probe 5	F	CGGTTGAGGGAGTGT
MICB probe 5	R	ACACTCCCTCAACCG
MICB probe 6	F	TATTGGGAGAAAAACC
MICB probe 6	R	GGTTTTTCTCCCAATA
MICA probe 6 mut	F	TCAAAGGGAGGCTAAGGAGA
MICA probe 6 mut	R	TCTCCTTAGCCTCCCTTTGA
MICB probe 6 mut	F	TATTGGGCTAAAAACC
MICB probe 6 mut	R	GGTTTGCAGATCAATA

Supplementary Table 6 Flow cytometry antibodies

Antibody	Manufacturer & Catalog	Website containing information of specificity and validation
PE anti-human CD147 (TRA-1-85) clone HIM6	Biolegend 306211	https://www.biolegend.com/en-us/products/pe-anti-human-cd147-antibody-16272
Brilliant Violet 421 anti-human PD-L1 clone 29E.2A3	Biolegend 329713	https://www.biolegend.com/en-us/products/brilliant-violet-421-anti-human-cd274-b7-h1-pd-l1-antibody-7261
APC anti-human HLA-E clone 3D12	Biolegend 342605	https://www.biolegend.com/en-us/products/apc-anti-human-hla-e-antibody-10760
PE-Cyanine7 anti-human LAP(TGF-B1) clone S20006A	Biolegend 300007	https://www.biolegend.com/en-us/products/pe-cyanine7-anti-human-lap-tgf-beta1-antibody-21121
eFluor506 anti-human CD45 clone HI30	eBioscience 69-0459-42	https://www.thermofisher.cn/cn/en/antibody/product/CD45-Antibody-clone-HI30-Monoclonal/69-0459-42
FITC anti-human CD3 clone HIT3a	Biolegend 300306	https://www.biolegend.com/en-us/products/fitc-anti-human-cd3-antibody-751
Super Bright 436 anti-human CD56 clone TULY56	eBioscience 62-0566-42	https://www.thermofisher.cn/cn/en/antibody/product/CD56-NCAM-Antibody-clone-TULY56-Monoclonal/62-0566-42
PE anti-human Granzyme B clone GB11	eBioscience 12-8899-41	https://www.thermofisher.cn/cn/en/antibody/product/Granzyme-B-Antibody-clone-GB11-Monoclonal/12-8899-41
PE-Dazzle 594 anti-human/mouse Granzyme B clone QA16A02	Biolegend 372216	https://www.biolegend.com/en-us/products/pedazzle-594-anti-humanmouse-granzyme-b-recombinant-antibody-15598
APC anti-human Perforin clone DG9	eBioscience 17-9994-42	https://www.thermofisher.cn/cn/en/antibody/product/Perforin-Antibody-clone-dG9-delta-G9-Monoclonal/17-9994-42
PerCP-Cyanine 5.5 anti-human IFN gamma clone 4S.B3	eBioscience 45-7319-42	https://www.thermofisher.cn/cn/en/antibody/product/IFN-gamma-Antibody-clone-4S-B3-Monoclonal/45-7319-42
PE-Cyanine7 anti-human TNF alpha clone MAb11	eBioscience 25-7349-82	https://www.thermofisher.cn/cn/en/antibody/product/TNF-alpha-Antibody-clone-MAb11-Monoclonal/25-7349-82
PE anti-human CD107a clone eBioH4A3	eBioscience 12-1079-42	https://www.thermofisher.cn/cn/en/antibody/product/CD107a-LAMP-1-Antibody-clone-eBioH4A3-Monoclonal/12-1079-42

Alexa Fluor 700<br>anti-mouse CD45<br>clone 30-F11	eBioscience<br>56-0451-82	https://www.thermofisher.cn/cn/en/antibody/product/CD45-Antibody-clone-30-F11-Monoclonal/56-0451-82
Brilliant Violet 510<br>anti-mouse/human CD11b<br>clone M1/70	Biolegend<br>101263	https://www.biolegend.com/en-us/products/brilliant-violet-510-anti-mouse-human-cd11b-antibody-7993
Brilliant Violet 421<br>anti-mouse F4/80<br>clone BM8	Biolegend<br>123137	https://www.biolegend.com/en-us/products/brilliant-violet-421-anti-mouse-f4-80-antibody-7199
BUV395<br>anti-mouse Ly-6G<br>clone 1A8	BD<br>565964	https://www.bdbiosciences.com/zh-cn/products/reagents/flow-cytometry-reagents/research-reagents/single-color-antibodies-ruo/buv395-rat-anti-mouse-ly-6g.565964
PE/Dazzle 594<br>anti-mouse CD11c<br>clone N418	Biolegend<br>117347	https://www.biolegend.com/en-us/products/pe-dazzle-594-anti-mouse-cd11c-antibody-9846
PerCP-Cyanine5.5<br>anti-mouse Ly-6C<br>clone HK1.4	eBioscience<br>45-5932-80	https://www.thermofisher.cn/cn/en/antibody/product/Ly-6C-Antibody-clone-HK1-4-Monoclonal/45-5932-80
Brilliant Violet 510<br>anti-mouse CD4<br>clone GK1.5	Biolegend<br>100449	https://www.biolegend.com/en-us/products/brilliant-violet-510-anti-mouse-cd4-antibody-10707
Brilliant Violet 605<br>anti-mouse CD8a<br>clone 53-6.7	Biolegend<br>100744	https://www.biolegend.com/en-us/products/brilliant-violet-605-anti-mouse-cd8a-antibody-7636
PE<br>anti-mouse NK-1.1<br>clone PK136	Biolegend<br>108707	https://www.biolegend.com/en-us/products/pe-anti-mouse-nk-1-1-antibody-431
Brilliant Violet 711<br>anti-mouse CD3<br>clone 17A2	Biolegend<br>100241	https://www.biolegend.com/en-us/products/brilliant-violet-711-anti-mouse-cd3-antibody-10022
APC anti-mouse CD107a<br>(LAMP-1)<br>clone 1D4B	Biolegend<br>121613	https://www.biolegend.com/en-us/products/apc-anti-mouse-cd107a-lamp-1-antibody-6081
Brilliant Violet 605<br>anti-mouse CD206 (MMR)<br>clone C068C2	Biolegend<br>141721	https://www.biolegend.com/en-us/products/brilliant-violet-605-anti-mouse-cd206-mmr-antibody-8729
Brilliant Violet 711<br>anti-mouse CD274 (PD-L1)<br>clone 10F.9G2	Biolegend<br>124319	https://www.biolegend.com/en-us/products/brilliant-violet-711-anti-mouse-cd274-b7-h1-pd-l1-antibody-9808
Brilliant Violet 421<br>anti-mouse FOXP3<br>clone MF-14	Biolegend<br>126419	https://www.biolegend.com/en-us/products/brilliant-violet-421-anti-mouse-foxp3-antibody-12143

Alexa Fluor 488<br>anti-mouse CD366 (TIM3)<br>clone 8B.2C12	eBioscience<br>53-5871-80	https://www.thermofisher.cn/cn/en/antibody/product/CD366-TIM3-Antibody-clone-8B-2C12-Monoclonal/53-5871-80
Brilliant Violet 650<br>anti-Mouse CD279 (PD-1)<br>clone J43	BD<br>744546	https://www.bdbiosciences.com/zh-cn/products/reagents/flow-cytometry-reagents/research-reagents/single-color-antibodies-ruo/bv650-hamster-anti-mouse-cd279-pd-1.744546
PE-Cyanine7<br>anti-mouse IFN gamma<br>clone XGM1.2	Biolegend<br>505825	https://www.biolegend.com/en-us/products/pe-cyanine7-anti-mouse-ifn-gamma-antibody-5865
PE<br>anti-human MICA/B<br>clone 6D4	eBioscience<br>12-5788-42	https://www.thermofisher.cn/cn/en/antibody/product/MICA-B-Antibody-clone-6D4-Monoclonal/12-5788-42

*The antibodies listed in this table were used at a dilution of 1:50.

Reference:

1. Barrow, A. D. *et al.* Natural Killer Cells Control Tumor Growth by Sensing a Growth Factor. *Cell* **172**, 534-548.e19 (2018).
