# Supplementary material for: Circular EZH2-encoded EZH2-92aa mediates immune evasion in glioblastoma via inhibition of surface NKG2D ligands
Source: Nat Commun. 2022 Aug 15;13:4795. doi: 10.1038/s41467-022-32311-2 (PMC9378736; doi:10.1038/s41467-022-32311-2)
Supplement: Supplementary file 3 — Description of Additional Supplementary Files [file 41467_2022_32311_MOESM3_ESM.pdf]

Supplementary Data 1: **Genes differentially expressed between GBM and adjacent brain tissues of 12 paired samples**. Table listing the differentially expressed genes (DEGs) between tumour and adjacent tissues in 12 paired GBM samples. A total of 2,289 differentially expressed circRNAs were identified, and 984 of which were upregulated in tumour samples. DEGs were selected based on a log2 fold-change ≥ 1 and FDR < 0.05.

Supplementary Data 2: **Identified circORFs with encoding potential**. ORFs were annotated in the tumour-upregulated circRNAs. A total of 473 circORFs with junction-spanning sequences were identified in this list.

Supplementary Data 3: **Pathway analysis based on the RNA-seq data of 12 paired GBM samples.** GSEA was performed based on the RNA-seq data of 12 paired GBM samples. The table lists enriched pathways significantly correlated with circEZH2.
