# Supplementary material for: Circular EZH2-encoded EZH2-92aa mediates immune evasion in glioblastoma via inhibition of surface NKG2D ligands
Source: Nat Commun. 2022 Aug 15;13:4795. doi: 10.1038/s41467-022-32311-2 (PMC9378736; doi:10.1038/s41467-022-32311-2)
Supplement: Supplementary file 7 — Reporting Summary [file 41467_2022_32311_MOESM7_ESM.pdf]

nature portfolio

Corresponding author(s): Xiuxing Wang, Yu Shi, Nu Zhang

Last updated by author(s): Aug 7, 2022

Reporting Summary

Nature Portfolio wishes to improve the reproducibility of the work that we publish. This form provides structure for consistency and transparency in reporting. For further information on Nature Portfolio policies, see our Editorial Policies and the Editorial Policy Checklist.

Statistics

For all statistical analyses, confirm that the following items are present in the figure legend, table legend, main text, or Methods section.

n/a	Confirmed	
☐	☒	The exact sample size (*n*) for each experimental group/condition, given as a discrete number and unit of measurement
☐	☒	A statement on whether measurements were taken from distinct samples or whether the same sample was measured repeatedly
☐	☒	The statistical test(s) used AND whether they are one- or two-sided<br>*Only common tests should be described solely by name; describe more complex techniques in the Methods section.*
☒	☐	A description of all covariates tested
☐	☒	A description of any assumptions or corrections, such as tests of normality and adjustment for multiple comparisons
☐	☒	A full description of the statistical parameters including central tendency (e.g. means) or other basic estimates (e.g. regression coefficient) AND variation (e.g. standard deviation) or associated estimates of uncertainty (e.g. confidence intervals)
☐	☒	For null hypothesis testing, the test statistic (e.g. *F*, *t*, *r*) with confidence intervals, effect sizes, degrees of freedom and *P* value noted<br>*Give P values as exact values whenever suitable.*
☒	☐	For Bayesian analysis, information on the choice of priors and Markov chain Monte Carlo settings
☒	☐	For hierarchical and complex designs, identification of the appropriate level for tests and full reporting of outcomes
☒	☐	Estimates of effect sizes (e.g. Cohen's *d*, Pearson's *r*), indicating how they were calculated

Our web collection on statistics for biologists contains articles on many of the points above.

Software and code

Policy information about availability of computer code

Data collection	ZEISS Zen (black edition version 2), QuantStudio 5 Operating Software (version 1.4.0), FACS Diva software (version 7)
Data analysis	RSEM (version 1.2.19), fastp (version 0.18.0), TopHat2 (version 2.1.1), Bowtie2 (version 2.3.0), find_circ (version 1.2), R language (version 3.2.19), GraphPad Prism (version 8), FlowJo (version 10.6.2), ImageJ (version 1.52), NDP.view (version 2), Image Lab (version 6.0.1), xCell (version 2020), GEPIA (version2)

For manuscripts utilizing custom algorithms or software that are central to the research but not yet described in published literature, software must be made available to editors and reviewers. We strongly encourage code deposition in a community repository (e.g. GitHub). See the Nature Portfolio guidelines for submitting code & software for further information.

Data

Policy information about availability of data

All manuscripts must include a data availability statement. This statement should provide the following information, where applicable:

- Accession codes, unique identifiers, or web links for publicly available datasets
- A description of any restrictions on data availability
- For clinical datasets or third party data, please ensure that the statement adheres to our policy

The sequencing data were deposited in the NCBI database under the accession ID PRJNA525736 [https://www.ncbi.nlm.nih.gov/bioproject/525736] and PRJNA862279 [https://www.ncbi.nlm.nih.gov/bioproject/862279]. The raw clinical data of glioma patients (containing personal information including names, record

numbers and contacts, etc.) are protected and are not available due to data privacy laws. However, the clinical data with personal information removed are available and provided within the Source Data file, covering information including the expression levels of EZH2-92aa and gene mutation status, etc. The remaining data are available within the Article, Supplementary Information, Source Data file. Source data are provided with this paper.

Human research participants

Policy information about studies involving human research participants and Sex and Gender in Research.

Reporting on sex and gender	Sex and gender were identified as insignificant factor in prognosis analysis. Source data disaggregated for sex has been collected and provided within the Source Data file. All pathologically diagnosed glioma samples and adjacent normal brain tissues used in this study were collected from the Department of Neurosurgery of the 1st Affiliated Hospital of Sun Yat-sen University with informed consent.
Population characteristics	63 Frozen human glioma samples (31 males and 32 females, aged from 19 to 68) were used. No personal information about the identity of the patients was made accessible to the researchers.
Recruitment	63 human glioma frozen samples were collected with patient consent from the biobank of the Department of Neurosurgery of the First Affiliated Hospital of Sun Yat-sen University. No self-selection bias was anticipated.
Ethics oversight	The study was approved by the Ethics Institutional Review Boards of the First Affiliated Hospital of Sun Yat-sen University (Approval No. [2020]322) and complied with all relevant ethical regulations regarding human participants.

Note that full information on the approval of the study protocol must also be provided in the manuscript.

Field-specific reporting

Please select the one below that is the best fit for your research. If you are not sure, read the appropriate sections before making your selection.

☒ Life sciences ☐ Behavioural & social sciences ☐ Ecological, evolutionary & environmental sciences

For a reference copy of the document with all sections, see nature.com/documents/nr-reporting-summary-flat.pdf

Life sciences study design

All studies must disclose on these points even when the disclosure is negative.

Sample size	For animal studies, sample size was chosen to comply with the 3R principles to minimize the number of mice used. For clinical data, sample size was determined based on our previous experience and the number of samples with available prognostic information. For sequencing data, sample size was determined based on the dataset used in our previous study (https://doi.org/10.1038/s41556-021-00639-4).
Data exclusions	Animals failing to grow tumour after intracranial injection of glioma stem cells were excluded.
Replication	All data were reliably reproduced. The number of repeats is indicated in each figure legend.
Randomization	For in vivo studies, all mice were randomly divided into each group without any self-selection before the start of each experiment. For clinical data analysis, all glioma samples were collected from patients who received no treatment before surgery. They were allocated to high- or low-expression group according to the expression of EZH2-92aa determined by semiquantitative western blot. Information of covariates such as sex, age, IDH1 mutation status and 1p19q co-deletion status was also collected, and a multivariate cox regression analysis was run to determine the prognostic value of EZH2-92aa, which is independent from aforementioned variates.
Blinding	For in vivo studies, investigators were not blinded to the animal experiments as ensuring a success intracranial glioma implantation required professional knowledge and experience. For sequencing data processing and quality control, the investigator in charge was blinded to sample condition. For clinical data analysis, the personal information of patients were encrypted before analysis.

Reporting for specific materials, systems and methods

We require information from authors about some types of materials, experimental systems and methods used in many studies. Here, indicate whether each material, system or method listed is relevant to your study. If you are not sure if a list item applies to your research, read the appropriate section before selecting a response.

Materials & experimental systems

n/a	Involved in the study
☐	☒ Antibodies
☐	☒ Eukaryotic cell lines
☒	☐ Palaeontology and archaeology
☐	☒ Animals and other organisms
☒	☐ Clinical data
☒	☐ Dual use research of concern

Methods

n/a	Involved in the study
☒	☐ ChIP-seq
☐	☒ Flow cytometry
☒	☐ MRI-based neuroimaging

Antibodies

Antibodies used

The following antibodies were used in this study:

For immunoblotting:
anti-flag (1:5,000, F1804, clone M2, Sigma-Aldrich), anti-EZH2 (1:1,000, 07-689, Merck Millipore), anti-H3K27me3 (1:1,000, 9733S, clone C36B11, Cell Signaling Technology), anti-Histone H3 (1:2,000, 4499S, clone D1H2, Cell Signaling Technology), anti-FBXW7 (1:1,000, ab109617, Abcam), anti-DDX3 (1:1,000, 11115-AP, Proteintech), anti-6xHis (1:1,000,ab18184, clone HIS.H8, Abcam), anti-HA (1:1,000, 35534, SAB), anti-β-tubulin (1:5,000, T5201, clone TUB2.1, Sigma-Aldrich), anti-β-actin (1:5,000, A1978, clone AC15, Sigma-Aldrich) and the custom rabbit polyclonal antibody specific for EZH2-92aa (1:500, produced by GenScript Biotech, Jiangsu, China); HRP-conjugated secondary antibodies: anti-rabbit IgG (1: 10,000, 5220-0336, SeraCare), anti-mouse IgG (1:10,000, 5220-0341, SeraCare)

For flow cytometry (diluted at 1:50):
PE anti-human CD147 (TRA-1-85) clone HIM6, Biolegend 306211
Brilliant Violet 421 anti-human PD-L1 clone 29E.2A3, Biolegend 329713
APC anti-human HLA-E clone 3D12, Biolegend 342605
PE-Cyanine7 anti-human LAP(TGF-B1) clone S20006A, Biolegend 300007
eFluor506 anti-human CD45 clone HI30, eBioscience 69-0459-42
FITC anti-human CD3 clone HIT3a, Biolegend 300306
Super Bright 436 anti-human CD56 clone TULY56, eBioscience 62-0566-42
PE anti-human Granzyme B clone GB11, eBioscience 12-8899-41
PE-Dazzle 594 anti-human/mouse Granzyme B clone QA16A02, Biolegend 372216
APC anti-human Perforin clone DG9, eBioscience 17-9994-42
PerCP-Cyanine 5.5 anti-human IFN gamma clone 4S.B3, eBioscience 45-7319-42
PE-Cyanine7 anti-human TNF alpha clone MAb11, eBioscience 25-7349-82
PE anti-human CD107a clone eBioH4A3, eBioscience 12-1079-42
PE anti-human MICA/B clone 6D4 eBioscience 12-5788-42
Alexa Fluor 700 anti-mouse CD45 clone 30-F11, eBioscience 56-0451-82
Brilliant Violet 510 anti-mouse/human CD11b clone M1/70, Biolegend 101263
Brilliant Violet 421 anti-mouse F4/80 clone BM8, Biolegend 123137
BUV395 anti-mouse Ly-6G clone 1A8, BD 565964
PE/Dazzle 594 anti-mouse CD11c clone N418, Biolegend 117347
PerCP-Cyanine5.5 anti-mouse Ly-6C clone HK1.4, eBioscience 45-5932-80
Brilliant Violet 510 anti-mouse CD4 clone GK1.5, Biolegend 100449
Brilliant Violet 605 anti-mouse CD8a clone 53-6.7, Biolegend 100744
PE anti-mouse NK-1.1 clone PK136, Biolegend 108707
Brilliant Violet 711 anti-mouse CD3 clone 17A2, Biolegend 100241
APC anti-mouse CD107a (LAMP-1) clone 1D4B, Biolegend 121613
Brilliant Violet 605 anti-mouse CD206 (MMR) clone C068C2, Biolegend 141721
Brilliant Violet 711 anti-mouse CD274 (B7-H1, PD-L1) clone 10F.9G2, Biolegend 124319
Brilliant Violet 421 anti-mouse FOXP3 clone MF-14, Biolegend 126419
Alexa Fluor 488 anti-mouse CD366 (TIM3) clone 8B.2C12, eBioscience 53-5871-80
Brilliant Violet 650 anti-Mouse CD279 (PD-1) clone J43, BD 744546
PE-Cyanine7 anti-mouse IFN gamma clone XGM1.2, Biolegend 505825

For other in vitro studies:
NKG2D blocking antibody (10µg/ml, BE0351, BioXcell), anti-BrdU antibody (2µg/tube, SC-32323, clone IIB5, Santa Cruz)

For in vivo studies:
anti-PD1 antibody (10 mg/kg, BE0146, BioXcell, diluted at 1:5 in PBS)

Validation

The EZH2-92aa antibody specifically recognizes the unique 10 amino-acid sequence of the C-terminus of EZH2-92aa (Supplementary Fig. 2b) and is validated for use in immunoblotting with knock-down cell lines as control, specific for human and murine EZH2-92aa (Supplementary Fig. 2f and 3b), and validated for use in ChIP with IgG as control (Supplementary Fig. 4h).

Other antibodies have been validated by their manufacturers, with detailed specificity described on their websites:
anti-flag (F1804, Sigma-Aldrich): recognizing N-Asp-Tyr-Lys-Asp-Asp-Asp-Asp-Lys-C; validated for WB, IP, IF, IHC [https://www.sigmaaldrich.cn/CN/en/product/sigma/f1804]
anti-EZH2 (07-689, Merck Millipore): recognizing EZH2; validated for WB, ChIP [https://www.sigmaaldrich.cn/CN/en/product/mm/07689]

anti-H3K27me3 (9733S, Cell Signaling Technology): recognizing endogenous levels of histone H3 only when tri-methylated on Lys27; validated for WB, IHC, IF, ChIP [https://www.cellsignal.com/products/primary-antibodies/tri-methyl-histone-h3-lys27-c36b11-rabbit-mab/9733?site-search-type=Products&N=4294956287&Ntt=9733s&fromPage=plp&_requestid=4391705&country=USA]
anti-Histone H3 (4499S, Cell Signaling Technology): recognizing endogenous levels of total Histone H3 protein, including isoforms H3.1, H3.2, and H3.3; validated for WB, IHC, IF [https://www.cellsignal.com/products/primary-antibodies/histone-h3-d1h2-xp-rabbit-mab/4499?site-search-type=Products&N=4294956287&Ntt=4499s&fromPage=plp&_requestid=4459769]
anti-FBXW7 (ab109617, Abcam): recognizing FBXW7; validated for WB, IF, IP, IHC [https://www.abcam.com/fbxw7-antibody-ab109617.html]
anti-DDX3 (11115-AP, Proteintech): recognizing DDX3; validated for WB, IP, IHC, IF, ELISA [https://www.ptgcn.com/products/DDX3-Antibody-11115-1-AP.htm]
anti-6xHis (ab18184, Abcam): recognizing His-tagged recombinant proteins; validated for WB, IP, IF [https://www.abcam.com/6x-his-tag-antibody-hish8-ab18184.html]
anti-HA (35534, SAB): recognizing transfected proteins containing HA-tag; validated for WB [https://www.sabbiotech.com.cn/g-8787-HA-Tag-Antibody-35534.html]
anti-β-tubulin (T5201, Sigma-Aldrich): recognizing all five isoforms of β-tubulin (β1-β5) and reacting with the β-Lc and β-Sc fragments in the carboxy-terminal part of β–tubulin in immunoblotting; validated for WB, IHC, IF [https://www.sigmaaldrich.cn/CN/en/product/sigma/t5201]
anti-β-actin (A1978, Sigma-Aldrich): recognizing an epitope located on the N-terminal end of the β-isoform of actin; validated for WB, IHC, IF [https://www.sigmaaldrich.cn/CN/en/product/sigma/a1978]
For flow cytometry antibodies, the information is summarized in Supplementary Table 6.

Eukaryotic cell lines

Policy information about cell lines and Sex and Gender in Research

Cell line source(s)	HEK293T ATCC CRL-3216<br>NK-92MI ATCC CRL-2408<br>NHA Lonza CC-2565<br>Glioma stem cell lines including 387, 456, 4121, 3691, MES28, GSC23 were kindly provided by Dr. Jeremy N. Rich.
Authentication	Cells were authenticated using short tandem repeat (STR) fingerprinting where applicable.
Mycoplasma contamination	All cell lines were tested negative for mycoplasma.
Commonly misidentified lines<br>(See ICLAC register)	No commonly misidentified cell lines were used in this study.

Animals and other research organisms

Policy information about studies involving animals; ARRIVE guidelines recommended for reporting animal research, and Sex and Gender in Research

Laboratory animals	6 week-old female NOD.CB17-PrkdcscidIl2rgtm1/Bcgen (B-NDG; Prkdc(-/-), IL2rg(X-/X-)) mice and 6 week-old female C57BL/6 mice were used.
Wild animals	The study did not involve wild animals.
Reporting on sex	Sex has not been confirmed as a critical factor for glioma tumorigenesis and prognosis. Female mice were used in this study to establish orthotopic glioblastoma model for their tractability.
Field-collected samples	The study did not involve field-collected samples.
Ethics oversight	All animal experiments conducted in this study were approved by the Ethics Institutional Review Boards of the First Affiliated Hospital of Sun Yat-sen University (Approval No. [2021]171 and [2021]173).

Note that full information on the approval of the study protocol must also be provided in the manuscript.

Flow Cytometry

Plots

Confirm that:

☒ The axis labels state the marker and fluorochrome used (e.g. CD4-FITC).

☒ The axis scales are clearly visible. Include numbers along axes only for bottom left plot of group (a 'group' is an analysis of identical markers).

☒ All plots are contour plots with outliers or pseudocolor plots.

☒ A numerical value for number of cells or percentage (with statistics) is provided.

Methodology

Sample preparation	For in vitro studies, cells were collected at indicated time point after co-culture, washed and resuspended in FACS staining buffer.<br>For isolation of cells from mice, fresh mouse brain samples were cut into pieces and digested in DMEM supplemented with collagenase IV (1 mg/ml, Gibco), DNase I (20 U/ml, Sigma-Aldrich) and hyaluronidase (0.01%, Solarbio) for 30 minutes at 37° C. After digestion, the cells were filtered through a 70-µm strainer, and Debris Removal Solution (130-109-398, Miltenyi Biotec) was applied to remove myelin according to the manufacturer's instructions. Cell pellets were then treated with RBC lysis buffer (C3702, Beyotime) and resuspended in FACS staining buffer (PBS containing 2% FBS).<br>For CD107a and intracellular staining, cells were incubated with phorbol 12-myristate 13-acetate (PMA; 50 ng/ml, MedChemExpress), ionomycin (1 µg/ml, Sigma), monensin solution (00-4505-51, eBioscience), brefeldin A solution (00-4506-51, eBioscience) and PE-CD107a for 4 hours at 37°C in an incubator as previously reported.
Instrument	BD LSRFortessa X-20 or Cytek Aurora
Software	FACS Diva software (version 7), SpectroFlo (version 3) and FlowJo (version 10.6.2)
Cell population abundance	Post-sort fractions were >95% as determined by a post-sort purity check of representative samples.
Gating strategy	Gating strategy is provided in Supplementary Figure 3 and 7, with dead cells and non-singlets excluded:<br>human NK cells: CD45+CD3-CD56+<br>glioma stem cells from nude mice xenograft: TRA-1-85(CD147)+<br>murine PMN-MDSCs: CD45+CD11b+LY6G+<br>murine M-MDSCs: CD45+CD11b+F4/80-LY6G-CD11c-LY6C+<br>murine Macrophages: CD11b+F4/80+CD45hi<br>murine Microglia: CD11b+F4/80+CD45lo<br>murine NK cells: CD45+CD3-NK1.1+<br>murine CD8 T cells: CD45+CD3+CD8+<br>murine regulatory T cells: CD45+CD3+CD4+FoxP3+

☒ Tick this box to confirm that a figure exemplifying the gating strategy is provided in the Supplementary Information.
